# Molecular basis of the multifaceted functions of human leucyl-tRNA synthetase in protein synthesis and beyond

**DOI:** 10.1093/nar/gkaa189

**Published:** 2020-03-30

**Authors:** Ru-Juan Liu, Tao Long, Hao Li, JingHua Zhao, Jing Li, MingZhu Wang, Andrés Palencia, JinZhong Lin, Stephen Cusack, En-Duo Wang

**Affiliations:** 1 School of Life Science and Technology, ShanghaiTech University, 100 Haike Road, Shanghai 201210, P.R. China; 2 State Key Laboratory of Molecular Biology, CAS Center for Excellence in Molecular Cell Science, Shanghai Institute of Biochemistry and Cell Biology, Chinese Academy of Sciences; University of Chinese Academy of Sciences, 320 Yueyang Road, Shanghai 200031, P.R. China; 3 State Key Laboratory of Genetic Engineering, School of Life Sciences, Fudan University, Shanghai, P.R. China; 4 Institutes of Physical Science and Information Technology, Anhui University, Hefei, Anhui, 230601, P.R. China; 5 Institute for Advanced Biosciences (IAB), Structural Biology of Novel Drug Targets in Human Diseases, INSERM U1209, CNRS UMR 5309, University Grenoble Alpes, 38000 Grenoble, France; 6 European Molecular Biology Laboratory, 71 Avenue des Martyrs, CS 90181, 38042, Grenoble, Cedex 9, France

## Abstract

Human cytosolic leucyl-tRNA synthetase (hcLRS) is an essential and multifunctional enzyme. Its canonical function is to catalyze the covalent ligation of leucine to tRNA^Leu^, and it may also hydrolyze mischarged tRNAs through an editing mechanism. Together with eight other aminoacyl-tRNA synthetases (AaRSs) and three auxiliary proteins, it forms a large multi-synthetase complex (MSC). Beyond its role in translation, hcLRS has an important moonlight function as a leucine sensor in the rapamycin complex 1 (mTORC1) pathway. Since this pathway is active in cancer development, hcLRS is a potential target for anti-tumor drug development. Moreover, LRS from pathogenic microbes are proven drug targets for developing antibiotics, which however should not inhibit hcLRS. Here we present the crystal structure of hcLRS at a 2.5 Å resolution, the first complete structure of a eukaryotic LRS, and analyze the binding of various compounds that target different sites of hcLRS. We also deduce the assembly mechanism of hcLRS into the MSC through reconstitution of the entire mega complex *in vitro*. Overall, our study provides the molecular basis for understanding both the multifaceted functions of hcLRS and for drug development targeting these functions.

## INTRODUCTION

Aminoacyl-tRNA synthetases (AaRSs) catalyze the attachment of amino acids (AAs) to their cognate transfer RNAs (tRNAs) to provide aminoacyl-tRNAs for translation. This process of aminoacylation occurs in two steps: (i) the formation of the activated aminoacyl-adenylate intermediate (aa-AMP) and (ii) the transfer of the aminacyl moiety to the 3′-end of the tRNA (1,2). Some non-cognate AAs that are structurally similar to the cognate one, can be mis-activated or mis-charged to the tRNA by AaRSs (3). To correct mistakes of this kind, some AaRSs have evolved a proofreading (editing) activity to hydrolyze mis-activated AAs (pre-transfer editing) or mis-charged AA-tRNA (post-transfer editing) (4,5). AaRSs not only play crucial roles in yielding substrates for protein synthesis and thus maintenance of cell survival, but also exhibit other functions independent of their catalytic activity and are closely linked to human disease (6–8). Increasing evidence shows that these non-classical functions of eukaryotic AaRSs are normally endowed by additional domains and motifs appended during evolution, which make eukaryotic AaRSs multifunctional, and their structural study important (9–12).

Based on some structural and functional characteristics, AaRSs are divided into class I and class II AaRS (13). Leucyl-tRNA synthetase (LRS), a class I AaRS, is particularly large and complex. Structurally, LRS contains an aminoacylation domain, a tRNA binding domain, and an editing domain, known as connective peptide 1 (CP1). Several structures of full-length or partial LRSs have been determined from prokaryotes or archaea (14–18), however, there is no structural information for LRSs from eukaryotes except for the isolated CP1 domains (19), hindering the exploration of the multifaceted functions of eukaryotic LRSs.

In contrast to *Escherichia* coli LRS (*Ec*LRS, Uniprot: P07813), which contains 860 AA residues, human cytosolic LRS (hcLRS, Uniprot: Q9P2J5), with 1176 AA residues (Supplementary Figure S1), has non-canonical functions aside from its capability for aminoacylation (20,21). Our previous studies have characterized the aminoacylation and editing activities of hcLRS (22,23), and show that higher eukaryotic LRS the UNE-L domain does not affect the catalytic activity and is not involved in substrate binding (22). We also found that hcLRS can mis-activate and mis-charge several leucine analogs, including norvaline (Nva) (23). Recent work has also shown that hcLRS can add the leucyl-group to lysine residues in proteins (24).

Importantly, besides these canonical functions, hcLRS, as well as *Saccharomyces cerevisiae* LRS (*Sc*LRS), play a key role in the AAs-induced activation of the target of rapamycin (TOR) pathway through sensing intracellular levels of leucine (20,25). The mammalian TOR (mTOR) is a conserved kinase that controls cell growth, including connecting cellular metabolism to a diverse set of environmental and intracellular signals, such as AAs (26). HcLRS functions as the intracellular leucine sensor to modulate the activity of the mTOR complex 1 (mTORC1) by directly binding with RagD GTPase, the mediator of the AAs-dependent mTORC1 pathway (20,27).

Hyperactivity of the mTORC1 pathway is beneficial for tumorigenesis and growth (26,28). Thus, inhibitors of mTORC1 or deregulation of the mTORC1 pathway are potential cancer therapies (26,28). As the primary inhibitors of mTORC1, rapamycin and its analogs have been widely studied for use as anti-cancer agents. However, rapamycin only inhibits a partial function of mTORC1, and leads to resistance in cancer cells (29). Thus, it is desirable to develop novel molecules targeting the mTORC1 pathway for cancer treatment. HcLRS is overexpressed in many cancers such as colon cancers, floor of the mouth carcinoma, skin squamous cell carcinoma and acute myeloid leukemia (30). Due to this overexpression, hcLRS has been reported as a promising anti-cancer target (30–32). Compounds that target hcLRS are classified into two classes: (i) leucinol or leucyl-adenylate sulfamate surrogates (LeuAMS) that target the aminoacylation active site of hcLRS; (ii) compounds that target the RagD binding site of hcLRS. Furthermore, the editing active site of LRSs from pathogens has proven to be an excellent target for the development of antimicrobial agents, e.g. Tavaborole, which treat fungal infections of the nail and nail bed (33). Many derivatives of tavaborole have shown promising results in treating other microbial infections such as gram-negative bacteria, TB and parasites (33–37). However, some of those compounds could also target human LRS. Thus, a high-resolution structure of hcLRS could be crucial in drug discovery not only for hcLRS targeting, but also avoiding unwanted side effects in the design of antimicrobial agents targeted to pathogenic LRSs.

In mammals, nine AaRSs: RRS, DRS, QRS, EPRS, IRS, LRS, KRS, MRS, together with three scaffold proteins: AaRS-interacting multi-functional proteins, AIMP1, AIMP2 and AIMP3, assemble into a multi-aminoacyl-tRNA synthetase complex (MSC). The assembly of the MSC plays a crucial role in translation (38,39), and regulates the non-canonical functions of these enzymes and cellular processes (40). MSC is thought to contain two stable subcomplexes connected via the scaffold AIMP2: subcomplex I contains EPRS, IRS, LRS, AIMP3 and MRS, while subcomplex II contains RRS, QRS and AIMP1 (38). Although the structures of the binary complex KRS/AIMP2 (41,42) and subcomplex II (43) are available, there is currently no high-resolution structure of the MSC. Based on previous studies, an architecture model of human MSC except for LRS and IRS was generated by Cho et al. (Supplementary Figure S2) (44).

To give insight into the above mentioned roles of hcLRS, we determined its crystal structure at a resolution of 2.5 Å. HcLRS is a complex protein with ten functional domains. Based on the structure, we analyzed the binding of available small molecules targeted to hcLRS. To address the question of how LRS participates in the MSC complex, we assayed the interaction capability of hcLRS with every protein subunit in subcomplex I and reconstituted the human MSC *in vitro*. We showed that hcLRS is incorporated into the MSC via a direct interaction with IRS. Our structural and biochemical studies thus give new insight into the biological functions of hcLRS and rational drug design against LRSs.

## MATERIALS AND METHODS

### Materials

Tris–HCl, tryptone, yeast extract, isopropyl-D-thiogalactoside (IPTG), bovine serum albumin (BSA), MgCl_2_, NaCl, Ethylene diamine tetraacetic acid (EDTA), dithiothreitol (DTT), guanosine monophosphate (GMP), adenosine triphosphate (ATP), CTP, GTP and UTP were purchased from Sangon Biotech (Shanghai, China). 3M sodium acetate (NaAc) solution (pH 5.2) were purchased from Sigma-Aldrich Co. LLC (St Louis, MO, USA). DNA fragment rapid purification and plasmid extraction kits were purchased from Yuanpinghao Biotech (Tianjing, China). KOD-plus mutagenesis kit and KOD-plus-neo Kit were from TOYOBO (Osaka, Japan). T4 DNA ligase, and all restriction endonucleases were obtained from Thermo Scientific (Waltham, MA, USA). [^3^H] L-leucine were purchased from Perkin Elmer Inc. (Waltham, MA, USA). Oligonucleotide primers were synthesized by Biosune (Shanghai, China). *Escherichia coli* Rosetta (DE3) cells were purchased from TIANGEN (Beijing, China). Nickel-nitrilotriacetic (Ni-NTA) Superflow resin was purchased from Qiagen, Inc. (Hilden, Germany). The Superdex™ 200 increase (10/300 GL) and Superose 6 increase (10/300 GL) were purchased from GE Healthcare (Fairfield, CT, USA). Crystallization kits were from Hampton research (Aliso Viejo, CA, USA). T7 RNA polymerase was purified from an overproduction strain in our laboratory (45).

### Preparation of tRNA

The tRNA^Leu^(CAG) genes were inserted into pUC19 to construct pUC19- tRNA^Leu^(CAG). Then, the DNA template was amplified for transcription using KOD-plus-neo polymerase with the forward primer (5′- TAATACGACTCACTATAGTCAGGATGGCCGAGCGGTCTA-3′) and reverse primer (5′-TGGTGTCAGGAGTGGGATTCGAACCCAC-3′). The tRNA was produced using *in vitro* T7 RNA polymerase transcription, as described previously (22,45). The tRNA concentration was determined using UV absorbance at 260 nm, and the molar absorption coefficient was calculated according to the sequence of tRNA (46).

### Protein expression and purification

The recombinant plasmid, pET16b-hcLRS was describe previously (47). The pET16b-hcLRS truncations were generated by KOD-plus mutagenesis kit. LRS and its mutants were purified by affinity chromatography on Ni-NTA Superflow resin and gel filtration as describe earlier (22,23). The protein concentrations were determined using UV absorbance at 280 nm, and the molar absorption coefficient was calculated according to the sequence of each protein (48). The human cytosolic MRS, IRS, EPRS, RRS, KRS, QRS, DRS, AIMP1, AIMP2 and AIMP3 genes were cloned from the cDNA of HEK293 cells. The genes that encode EPRS, RRS, IRS, QRS, DRS and AIMP1 were sub-cloned into a modified pET28b vector that expressed an 8×His-tag at the N- or C-terminus of each protein. The genes encoding KRS-AIMP2 and MRS-AIMP3 were sub-cloned into a modified pETDuet-1 vector for co-expression; an 8×His-tag was expressed at the N-terminus of KRS and MRS, and an additional Flag-tag was introduced at the C-terminus of MRS using the KOD-plus mutagenesis kit; AIMP2 and AIMP3 did not carry a tag. EPRS, RRS, IRS, QRS, DRS, AIMP1, KRS-AIMP2 and MRS-AIMP3 were all separately purified by Ni-NTA affinity chromatography and gel filtration.

### Aminoacylation activity determination and IC_50_ assay

The time course curve for aminoacylation by hcLRS for human cytosolic tRNA^Leu^(CAG) was determined at 37°C in a 25-μl mixture containing 20 mM Tris–HCl (pH 7.5), 10 mM NaCl, 15 mM MgCl_2_, 2 mM DTT, 2 mM ATP, 1 mg/ml BSA, 40 μM [^3^H]-Leu and 10 μM of tRNA^Leu^(CAG); The reaction was initiated upon addition of 4 nM enzyme. Aliquots (5 μl) of the reaction mixtures were removed at time intervals between 2 and 8 min, quenched on Whatman glass-fiber filter discs, soaked in 5% TCA and counted in PPO/POPOP/toluene.

To determine the half-maximal inhibitory concentration (IC_50_) of tavaborole to hcLRS, at least six concentrations of tavaborole were tested in aminoacylation activity of 5 nM hcLRS under the above reaction conditions. Data were fitted to a dose-response curve using GraphPad Prism.

### Protein crystallization, structure determination and refinement

Crystallization was performed at 16°C, using the hanging drop vapor diffusion method. For crystallization, LRS was concentrated to 8mg/ml. Protein solution (1 μl) was mixed with an equal volume of the reservoir solution, consisting of 20% (w/v) PEG 6000, 100 mM Tris–Cl, pH 8.0 and 200 mM Lithium chloride. The crystals were frozen in liquid nitrogen after transferring for a few seconds in the mother liquid which contained 15% glycerol (v/v) as a cryoprotectant.

All crystal diffraction data sets were collected at the Shanghai Synchrotron Radiation Facility beamlines (SSRF, Shanghai, China) BL-19U1 and BL-17U1. The diffraction data were processed using the HKL2000 program package (49). Further data analysis was performed with the CCP4 suite (50). The structure of LRS was initially solved by molecular replacement using PHASER (51) with the structure of aminoacylation domain and C-terminal domain of *Pyrococcus horikoshii* LRS (PDB ID: 1WZ2) and the structure of isolated hcLRS-CP1 as starting models, and was further improved by manual adjustments using COOT (52). Next, the model was refined using REFINE program in the PHENIX suite (53). The quality of the final model was evaluated using MOLPROBITY (http://molprobity.biochem.duke.edu/). Figures were drawn using PyMOL (http://www.pymol.org/). A structure-based multiple amino acid sequence alignment of LRSs from model organisms was generated using ESPript (54). The parameters for data collection and structure refinements are shown in Supplementary Table S1. The PDB IDs are 6LPF and 6LR6.

### *In vitro* assembly of human MSC

Purified human cytosolic LRS, EPRS, RRS, IRS, QRS, DRS, AIMP1, KRS-AIMP2 and MRS-AIMP3 proteins were mixed together and incubated for 2 h. The MSC complex was pulled down and purified further by anti-Flag beads (only MRS has the Flag tag). The quality of the complex was checked by sodium dodecyl sulphate-polyacrylamide gelelectrophoresis and gel-filtration. For the protein–protein interaction assays of hcLRS with every protein subunit of subcomplex I (IRS, MRS-AIMP3 and EPRS), purified hcLRS were incubated with each of them separately for 4 h and then run on a gel filtration to check for the formation of the protein complex, parallel incubation of protein with buffer were set as controls.

## RESULTS

### Overall structure of hcLRS

Full length hcLRS (FL-hcLRS, Supplementary Figure S1) which contains 1176 amino acids (AA) could be crystallized but the crystals only diffracted to 10 Å resolution. To improve diffraction quality, we decided to truncate the C-terminal UNE-L domain, which is not required for enzymatic function or activation of the mTORC1 pathway (20,22). After screening truncations with different lengths, we found that deletion of the last 106 residues (N1071-1176C), designated as hcLRS_d106, gives improved crystals that diffract up to 2.5 Å resolution. The hcLRS_d106 protein retains aminoacylation activity to tRNA^Leu^ (Supplementary Figure S3). Thus, the crystal structure of hcLRS reported below lacks the UNE-L domain. The asymmetric unit contains two non-crystallographically related hcLRS molecules with differences in B-factors and subtle differences in orientation of some of the peripheral domains.

**Figure 1.**
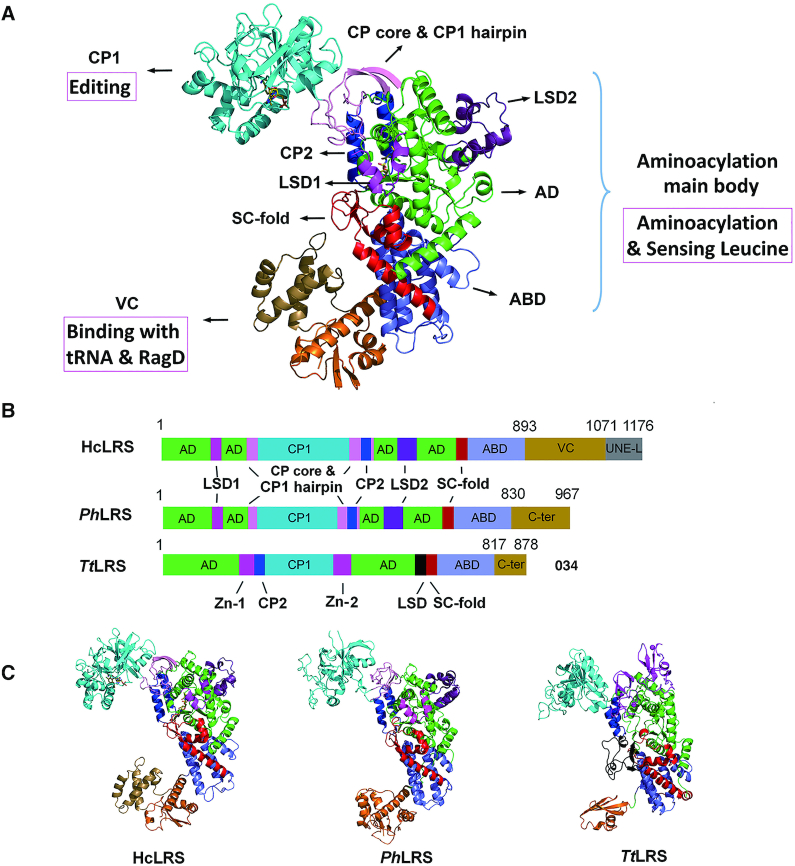
The overall structure of hcLRS. (**A**) Overall structure of hcLRS shown in cartoon. The Rossmann-fold domain (AD, green), the CP core & CP1 hairpin (pink), the CP1 editing domain (cyan), the CP2 domain (blue), the eukaryotic leucyl-specific domains 1 (magenta) and 2 (violet-purple), the SC-fold domain (red), the α-helix bundle domain (slate) and the VC domain (orange and sand); (**B**) A diagram shown the domains and motifs of hcLRS, *Ph*LRS and *Tt*LRS; (**C**) Structural comparison of hcLRS, *Ph*LRS and *Tt*LRS shown in cartoon from a same view.

The structure of hcLRS reveals a very complex modular architecture, which can be divided into nine domains/motifs including four main functional domains and five smaller insertion motifs (Figure 1A and B). The four main domains are: the Rossmann-fold aminoacylation domain (AD, shown in green in Figure 1A), the CP1 domain (shown in cyan in Figure 1A) for editing, the characteristic anticodon binding domain (ABD) for tRNA recognition (shown in slate in Figure 1A) and the vertebrate C-terminal (VC) domain for tRNA and RagD binding (shown in orange and sand in Figure 1A). The five smaller insertion domains/motifs are: the eukaryotic leucyl specific domains 1 (LSD1, residues 106–176, magenta) and 2 (LSD2, residues 606–659, violet-purple), the connective polypeptide 2 (CP2) domain (residues 534–569, blue) that are inserted into the AD, and the CP core and CP1 hairpin (residues 231–261, 510–535 and 570–575, pink) that links CP1, CP2 and AD; and the stem contact fold (SC-fold, residues 708–776, red) that connects the AD and ABD domains (Figure 1A and B). Taking the UNE-L domain into consideration, the FL-hcLRS contains ten domains/motifs in total (Figure 1B).

Architecturally, hcLRS resembles LRS from archaeon *P. horikoshii* (*Ph*LRS) more than those from bacteria (*E. coli* or *Thermus thermophilus, Ec*LRS *or Tt*LRS) (Figure 1C). HcLRS superposes well on *Ph*LRS except for the orientation of the CP core and CP1 hairpin, the CP1 domain and VC domain (Supplementary Figure S4). The main difference between bacterial and eukaryotic/archaeal LRSs comes from the insertion site of the CP1 editing domain (14–15,17). In bacterial LRS, the CP1 domain is inserted after CP2 between two zinc-fingers (Figure 1B and C). While in archaeal and eukaryotic LRS, the CP1 domain is inserted before CP2 domain through two long linkers (Figure 1B and C). For the structural comparison of each domains among hcLRS, *Ph*LRS and *Tt*LRS, the biggest difference comes from the fold of the C-terminal (VC for hcLRS, C-ter for *Ph*LRS and *Tt*LRS) domains (Figure 1B and C). Another noticeable differences come from the distinct orientations of the CP1 domain and the C-terminal domains.

**Figure 2.**
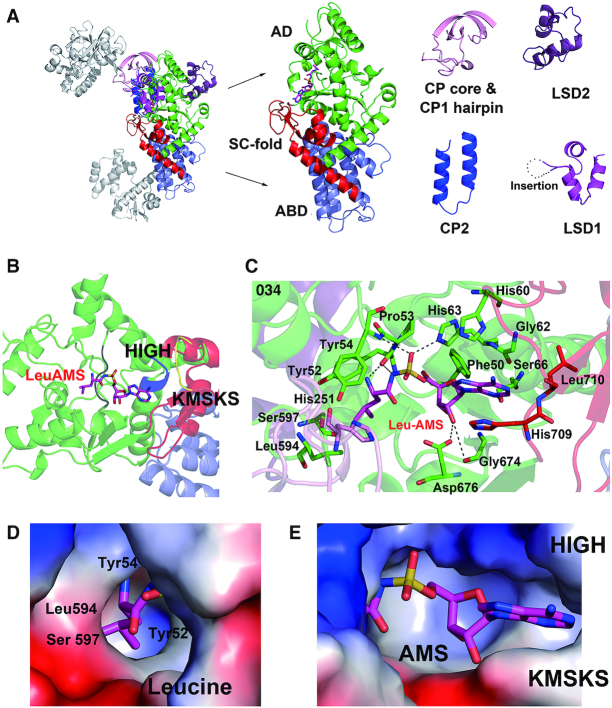
Aminoacylation active site of hcLRS and the binding details of LeuAMS. (**A**) The cartoon representation of aminoacylation main body of hcLRS, each inserted motifs are dissected from the structure for a clear view. (**B**) Shows HIGH motif (His 60 to His 63, blue) and KMSKS motif (Lys716 to Ser720, yellow), and the LeuAMS is shown in stick; (**C**) Binding details of the LeuAMS (stick representation) in the aminoacylation active site, residues within 4 Å are labeled. The binding pocket (electrostatics surface representation) of LeuAMS are shown separately for the Leucine moiety binding pocket (**D**) and AMS moiety binding pocket (**E**).

Based on function, the structure of hcLRS can be dissected into three main bodies (Figure 1A): (i) the aminoacylation main body which is comprised of the AD, ABD domain and all the five smaller insertion domains/motifs (Figure 2A); (ii) the CP1 editing domain; (iii) the VC domain that interacts with tRNA and RagD. We will now describe the structure of each main functional body and their related functions in more details.

### Aminoacylation main body and synthetic active site of hcLRS

The structure of AD domain, the ABD domain and the SC-fold motif are conserved in all class Ia AaRS (LRS, IRS and VRS) (Figure 2A). The AD domain exhibits a Rossmann-fold as in all class I AaRSs, the ABD domain is an α-helical bundle and the SC-fold motif linking the AD and ABD is composed of a characteristic β-α-α-β-α topology (Figure 2A). The structure of CP2, which is composed of two helices in hcLRS, is very similar to its equivalent in *Ph*LRS (16) (Figure 2A). For the specific domains, LSD1 has a long insertion (Arg106 to Ser154) when compared with LSD1 in *Ph*LRS (Figure 2A and Supplementary Figure S1). The primary sequence of LSD1 is of low complexity with repeated Glu and Lys residues and is conserved in all the eukaryotic LRSs (Supplementary Figure S1), and residues Pro115-Ala151 in this region were predicted to be structurally disordered (55). In our structure, only the first thirteen AA residues are visible in the electron density, suggesting that the rest of this region is structurally disordered (Figure 2A). The function of this insertion remains unclear in eukaryotic LSD1. The LSD2, which is located on the reverse side to the aminoacylation active site, is composed of three short α-helices and its biological function is also unclear. The topology of the CP core and CP1 hairpin is similar to that in *Ph*LRS (Supplementary Figure S4); however, the CP core is too close to the AD domain. When superposed hcLRS to the *Ph*LRS–tRNA complex, the 3′-end of tRNA will clash with the CP core (Supplementary Figure S5), suggesting that the CP core will have to change orientation upon the binding of tRNA substrate.

LeuAMS, a stable analog of LeuAMP, was bound in the aminoacylation active site in our structure (Figure 2B and C). Besides the Rossmann fold domain, two characteristic HIGH and KMSKS motifs are conserved in all the class I AaRSs (Supplementary Figure S1). HIGH motif (colored in blue in Figure 2B) locates to the end of an α-helix, is in the aminoacylation active site and interacts with LeuAMS (Figure 2B and C). The KMSKS motif that is important for tRNA binding and for ATP binding, especially the phosphate groups, is located in the SC-fold motif and does not have a direct interaction with LeuAMS (Figure 2B and C). When dissecting the LeuAMS into two parts, leucine and AMS, we find that the AMS (AMP) moiety mainly interacts with residues from the HIGH motif and with residues near the KMSKS motif (Figure 2C). This is a relatively open pocket when compare to the binding pocket of leucine (Figure 2D and E). Leucine, instead binds to a deep and narrow pocket formed by residues Tyr52, Tyr54, Leu594 and Ser597 (Figure 2C and D). Interestingly, residue His251 from the CP core is within 4 Å of the leucine, and the N atom from the imidazole ring of His251 is only 2.6 Å from the—OH group of Tyr54 (Figure 2C) suggesting a role of His251 in leucine binding. Indeed, our previous mutagenesis studies showed that the hcLRS_H251D mutation loses amino acid activation activity (47).

### CP1 domain and the catalytic mechanism of editing

In the class Ia AaRSs (LRS, IRS and VRS), the CP1 domain contains the post-transfer editing active site. Structures of CP1 domains have been described extensively in the prior literature including the 3.2 Å crystal structure of an isolated hcLRS-CP1 domain (19). But several outstanding questions remain, particularly with regard to the catalytic mechanism of post-transfer editing including specific roles of the tRNA, bound water molecules and conserved active site residues; and with regard to the specificity of inhibitors targeting the CP1 catalytic core.

**Figure 3.**
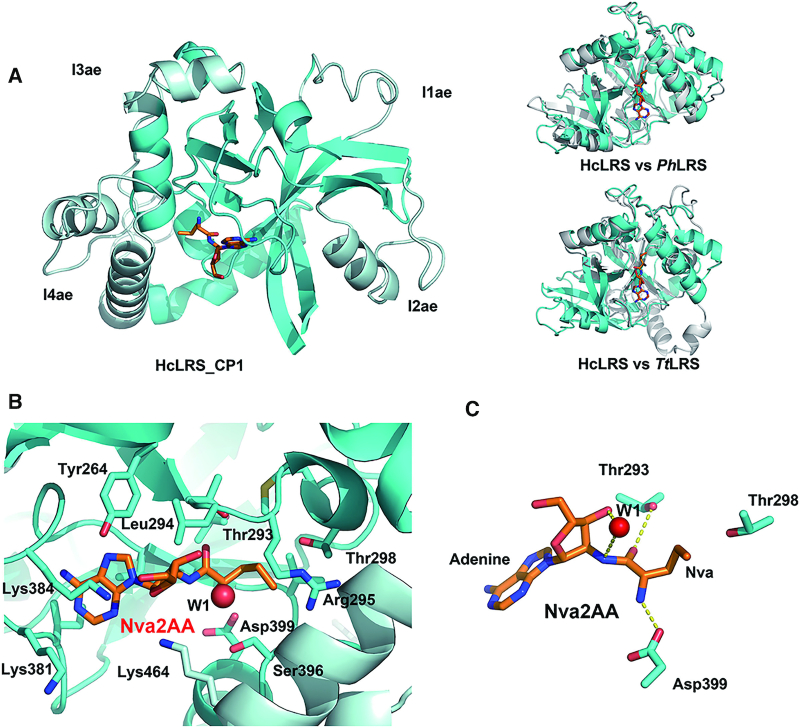
CP1 domain and editing active site of hcLRS. (**A**) Left panel is the cartoon representation of CP1 domain, the catalytic core is shown in cyan and the four insertions I1ae, I2ae, I 3ae and I4ae are shown in pale cyan; right panel is the CP1 domain superposition with *Ph*LRS-CP1(up) and TtLRS-CP1 (bottom); (**B**) Binding details of the Nva2AA (stick representation) in the editing active site, residues within 4 Å are labeled; (**C**) The catalytic crucial residues and a water molecule (sphere) that bound to 3′-OH together Nva2AA are shown in sticks.

The overall structure of CP1 in hcLRS is similar to the isolated CP1s, which presents as a globular β-barrel surrounded by α-helices (Figure 3A). The catalytic core (show in cyan in Figure 3A) of the editing domain is conserved in the CP1 domain of LRS, VRS and IRS. The structural differences of CP1 domain from hcLRS, *Ph*LRS and *Tt*LRS mainly come from the insertions (Figure 3A). Four insertions, namely I1ae, I2ae, I3ae and I4ae (show in pale cyan in Figure 3A) are inserted to peripheral surface of the catalytic core. Except for I4ae, the other three insertions locate far away from the editing active site. In our structure, a post-transfer editing analog Nva2AA (2′-(L-norvalyl) amino-2′-deoxyadenosine) is clearly bound at the editing active pocket. The residues involved in interaction with Nva2AA are mainly from the conserved ‘threonine-rich region’ (T-rich region) and the ‘GTG loop’ with one exception, Lys464, which is from a helix of I4ae insertion (Figure 3B). The ‘T-rich’ region and the ‘GTG loop’ are two characteristic motifs in the editing active site of the CP1 domain that are essential for editing activity (4,17,19).

For the catalytic mechanism, a novel ‘hybrid ribozyme/protein catalyst’ model was proposed on the basis of a molecular dynamic simulation study: editing is a self-cleavage reaction driven by tRNA^Leu^ in *Tt*LRS, and assisted by the T-rich region which stabilizes high-energy intermediates along the reaction path to improve the efficiency of editing (56). Experimentally, the role of the tRNA-A76 3′-OH group in the mechanism of LRS deacylation was tested in *E. coli* LRS by replaced the terminal adenosine of tRNA^Leu^ with 3′-deoxyadenosine, the results showed that the 3′-OH group of A76 has a prominent role in deacylation reaction (57). Interestingly, a very well ordered water molecule which hydrogen-bonds to the 3′-OH group of tRNA-A76 is observed in our structure (W1 in Figure 3B and C). This water molecule locates in an optimal position to act as the nucleophile for hydrolysis, and the distance between the oxygen atom of W1 (Ow atom) and the C atom (which is actually a N atom in Nva2AA to prevent hydrolysis) that links the Nva and adenosine being 3.4 Å (Figure 3C). It is possible that during deacylation in hcLRS, this water molecule W1, activated by the 3′-OH, acts as the nucleophile to attack the ester bond. Without knowing the exact role of each residue, the conserved catalytically important residues including Asp399 (equivalent to Asp345 of *Ec*LRS), Thr293 (Thr247 in *Ec*LRS) and Thr298 (Thr252 in *Ec*LRS) all locate near to Nva2AA (Figure 3B). The side chain of Asp399 forms a salt bridge with the NH_2_ group of Nva moiety, and the side chain of Thr293 hydrogen bonds to carbonyl oxygen atom of Nva moiety. Thr298, which is in the protruding direction of side chain moiety of Nva (Figure 3B), may contribute to control the size of editing pocket as revealed by it equivalent residue in bacterial LRS through biochemical assays (58,59). Our structure suggests that the 3′-OH group of tRNA-A76 and residues Asp399 and Thr298 from the protein are all involved in the post-transfer editing in hcLRS, which is in good agreement with the proposed ‘hybrid ribozyme/protein catalyst’ mechanism for post-transfer editing in bacterial LRSs.

### VC domain binding to tRNA^Leu^ and RagD

**Figure 4.**
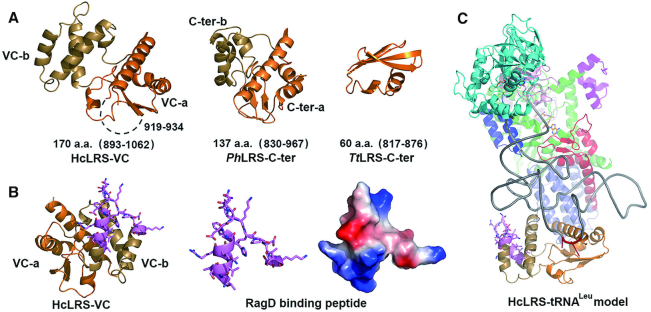
The structure of VC domain. (**A**) The cartoon representation of VC domain that composed of VC-a (orange) and VC-b (sand), and the structure of equivalent C-ter domains of *Ph*LRS and *Tt*LRS are shown for comparison. (**B**) Stick (left and middle) and electrostatics surface (right) representations of the RagD binding peptide of VC domain; (**C**) A proposed model of hcLRS–tRNA^Leu^ complex shown in cartoon, tRNA^Leu^ is shown in gray.

During evolution, the C-terminal domain of LRS is divergent (Supplementary Figure S1). In *Tt*LRS, the C-ter domain is a small domain which comprises only 60 AA residues (Figure 4A), while in hcLRS, the equivalent VC domain has 170 AA residues (893–1062, Figure 4A). The main function of the C-ter domain in bacteria and archaea LRS is to bind tRNA^Leu^. Since this domain is flexibly linked to the rest of LRS, it is generally invisible in the electron density map of LRS structures without bound tRNA (14,15). Luckily, in the current structure, the VC domain is visible even though there is no tRNA bound due to crystal contacts. The overall structure of the VC domain is different from the C-ter domain from *Ph*LRS or *Tt*LRS (Figure 4A). Structurally, it is composed of two discrete sub-domains. The first sub-domain comprises two β-strands and three a-helices adopting an α−β−α−β−α topology (colored in orange in Figure 4A), which we designate as the VC-a domain. The second, denoted VC-b domain, is composed of three helices (colored in sand in Figure 4A) and is inserted into the VC-a domain via two connecting peptides. Similarly, the C-ter domain of *Ph*LRS can be dissected into C-ter-a and C-ter-b sub-domains (Figure 4A). Structurally, the VC-a domain resembles the C-ter-a domain of *Ph*LRS (Figure 4A). In the *Ph*LRS complex with tRNA^Leu^ (PDB ID: 1WZ2), the tRNA only contacts residues from the C-ter-a domain and not the C-ter-b domain (16). Although the topology of the VC-b domain is similar to that of the C-ter-b domain, the length and orientation of the helices is different. By superposing the *Ph*LRS–tRNA complex onto hcLRS, a model can be obtained for the hcLRS–tRNA complex, which suggests that the VC domain orientation would have to change to avoid a clash with tRNA^Leu^. Indeed, if the VC domain orientation is adjusted by superimposing the VC-a domain onto the C-ter-a domain of *Ph*LRS, tRNA^Leu^ can be accommodated by the VC of hcLRS without clash. This docking model suggests that, as in *Ph*LRS-tRNA^Leu^, the long variable loop of tRNA^Leu^ only interacts with the VC-a domain but not the VC-b domain (Supplementary Figure S6).

Interestingly, RagD also binds to the VC domain of hcLRS. *In vitro* biochemical assays show that tRNA^Leu^ competes with RagD for hcLRS binding (20), suggesting that tRNA and RagD have exclusive access to hcLRS. What is the structural basis for this? A previous report showed that hcLRS residues 951–971 (colored in violet in Figure 4B) form the RagD-binding site. This RagD-binding peptide forms a helix-loop structure located in the VC-b domain (Figure 4B). In the proposed docking model for hcLRS–tRNA complex, the RagD binding peptide does not have direct contact with the tRNA (Figure 4C). However, the closest distance between RagD-binding peptide and tRNA main chain is 8 Å (Figure 4C) suggesting that when the tRNA is bound to hcLRS, there will be steric hindrance for simultaneous RagD binding, and *vice versa*.

Our results suggest that the binding of tRNA and RagD by hcLRS are via different regions from two discrete domains of the VC domain. The more conserved VC-a domain is mainly for binding with the long variable loop of tRNA^Leu^, while the less conserved VC-b domain gains the new function to bind RagD.

### Structural basis for binding of compounds targeting to hcLRS

HcLRS directly binds to RagD, the mediator of amino acid signaling to mTORC1, by sensing intracellular leucine concentration, resulting in the activation of the mTORC1 pathway (20). Indeed, leucine appears to be the master controller in the amino acid-dependent activation of the mTORC1 pathway (60). At present, there are some reported compounds that serve as potential anti-cancer agents, which target to hcLRS to inhibit leucine-dependent activation of mTORC1 pathway. The compounds target either the leucine or LeuAMP binding site or the RagD binding site to deregulate the mTORC1 pathway (27,30–32,61–63).

### Compounds targeting to the aminoacylation active site

**Figure 5.**
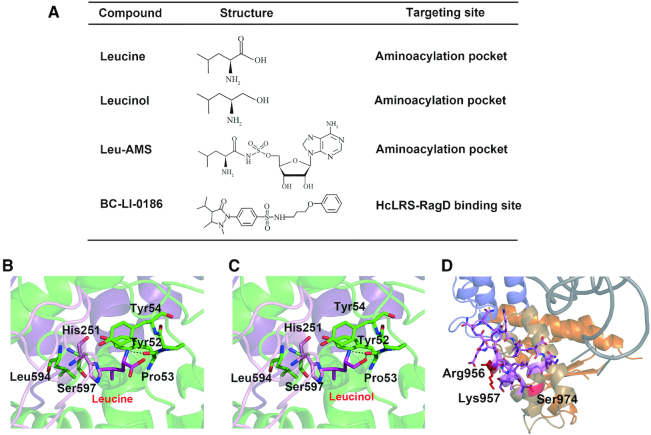
The representative compounds targeting to hcLRS. (**A**) The compound name, structure and targeting site are listed; the binding of leucine (**B**) and leucinol (**C**) in the aminoacylation active site are generated based on the structure of hcLRS-LeuAMS; (**D**) The BC-LI-0186 binding site (red color) in the proposed hcLRS-tRNA^Leu^ model.

As a competitive inhibitor of LRS, leucinol (Figure 5A), together with its derivatives, block leucine-mediated activation of mTORC1 by inhibiting leucine-sensing (20,61,64). Importantly, a derivative of leucinol exhibited significant cytotoxicity when treated to rapamycin resistant human colon cancer cells (63). AA-AMPs and AA-AMSs have been well-studied as inhibitors of AaRS (65,66). Some LeuAMS derivatives with modified adenosine and leucine moieties exhibited good activity against mTORC1 pathway, and showed cytotoxicity to various cancer cells (30,32). Based on the structure of the binding pocket of leucine and adenosine (Figures 2 and 5B), we proposed a docking model for leucinol (Figure 5C). Both LeuAMS and leucinol occupy the binding pocket of leucine and make it impossible for LRS to accommodate leucine, thereby blocking the activation of mTORC1 pathway. It is worth noting that the IC_50_ values of leucinol or LeuAMS derivatives in inhibiting mTORC1 pathway were lower than IC_50_ values of inhibition the aminoacylation activity of LRS (20,30,32,61–63).

### Compounds targeting hcLRS to disrupt the interaction between hcLRS and RagD

A small-molecule inhibitor, BC-LI-0186, has been developed to directly interact with hcLRS and disrupt its interaction with RagD. BC-LI-0186 could inhibit leucine-dependent activation of mTORC1 in cells with high specificity (31) and shows an anticancer effect upon treatment of non-small cell lung cancer (NSCLC) in mouse models (27). Significantly, this compound does not affect the aminoacylation activity of LRS, suggesting that the binding of tRNA is not affected. To investigate how BC-LI-0186 binds to hcLRS, we tried to co-crystallize hcLRS with BC-LI-0186, however, no crystal would grow after adding BC-LI-0186. We found that some residues from the RagD binding peptides are in the crystal packing interface (Supplementary Figure S7). Therefore, a possible explanation is that BC-LI-0186 binding to the RagD binding region of LRS destroys the crystal packing. Previous mutational experiments showed that residues Arg956, Lys957 and Ser974 are involved in the binding of BC-LI-0186. In our structure, these residues are in or close to the RagD binding region (Figure 5D). In the proposed tRNA binding model, these residues locate on the opposite side to the tRNA binding surface, which may explain why binding of tRNA to hcLRS is not affected by BC-LI-0186 (Figure 5D).

### Rational drug design of antimicrobial agents targeting to LRS

**Figure 6.**
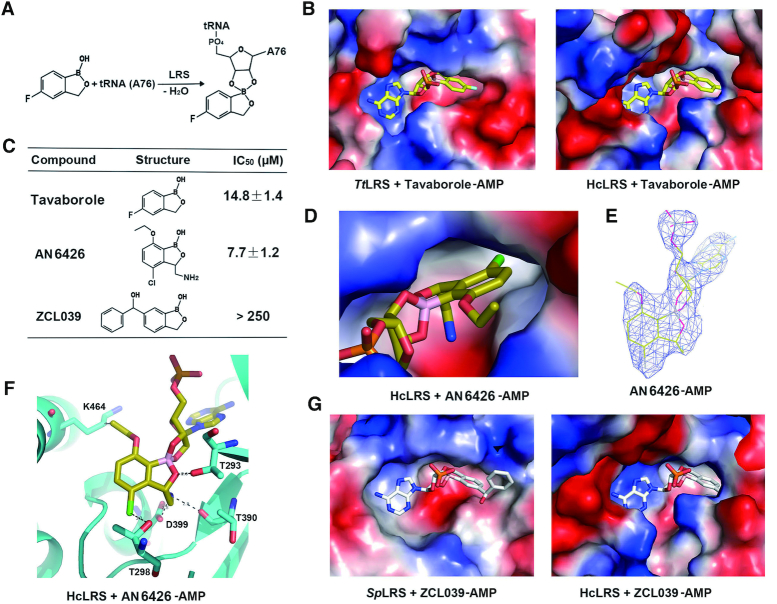
The inhibition effects of three anti-fungal or anti-bacteria benzoxaborole compounds (tavaborole, AN6426 and ZCL039) to hcLRS. (**A**) The reaction scheme between tavaborole and A76 of tRNA (or AMP) in LRS editing site; (**B**) Shows the binding pocket for tavaborole-AMP in *Tt*LRS and the docking model of tavaborole-AMP in hcLRS; (**C**) The IC_50_ values of tavaborole, AN6426 and ZCL039 to hcLRS assayed *in vitro*; The binding of AN6426–AMP in the complex structure of hcLRS–AN6426–AMP in electrostatics surface (**D**) and in cartoon (**F**); (**E**) The density map of AN6426–AMP in the complex structure of hcLRS–AN6426–AMP; (**G**) The binding pocket for ZCL039-AMP in *Sp*LRS, and the docking model of ZCL039-AMP in hcLRS-CP1 in which the ZCL-039 clashed with hcLRS-CP1.

Editing sites of LRSs from pathogenic microbes are excellent targets for the development of antimicrobial agents. The discovery of tavaborole led to other benzoxaborole derivatives possessing great potential in treating infections due to diverse bacterial, fungal or parasite pathogens (33,35,37). To avoid side effects from targeting human LRS, it is desirable to design compounds with high specificity to pathogenic LRSs but not to human LRSs. Due to the I4ae insertions, the editing active pockets size is smaller in archaeal and eukaryotic LRS than in bacterial LRS (18,19). Tavaborole is a small benzoxaborole that inhibits fungal LRS by binding in the amino acid part of the editing active site, covalently linking to A76 of tRNA^Leu^ and thus blocking the tRNA in the editing conformation (Figure 6A). When we superimpose the structure of hcLRS-CP1 to *Tt*LRS-CP1-tavaborole-A76 (tRNA), we found that tavaborole-A76 could be fitted into the editing pocket of hcLRS, which suggests that tavaborole may also have inhibition against hcLRS (Figure 6B). We then performed *in vitro* inhibition assay of tavaborole to hcLRS. The result showed that tavaborole inhibited the aminoacylation activity of hcLRS with an IC_50_ value of 14.8 μM (Figure 6C), suggesting that tavaborole indeed could target to hcLRS *in vitro*. However, the IC_50_ value for hcLRS is higher than those reported for fungal LRSs (2.1 μM for *Sc*LRS) (33), indicating that tavaborole have a higher specificity for fungal LRSs.

We were able to obtain a co-crystal structure of hcLRS bound with a tavaborole derivative, a previous reported anti-cryptosporidium benzoxaborole, AN6426 (37). The structure was refined to 3.0 Å resolution (Supplementary Table S1). The IC_50_ of AN6426 to hcLRS *in vitro* is 7.7 μM (Figure 6C). In the structure, AN6426-AMP is well accommodated in the editing pocket (Figure 6D and E) with the AN6426 moiety mainly binding to residues from the editing active site, such as Thr293, Thr298 and Asp399. The side chain of Lys 464 from the I4ae insertion is within the Van der Waals interaction distance with the ether group at the meta-C of AN6426 (Figure 6F). Structurally, this I4ae insertion makes the size of editing pocket from hcLRS much smaller than those from bacteria LRSs (Figure 3). One could imagine that if we added a bigger group to the meta-C of AN6426, this group will clash with the I4ae insertion of hcLRS. So it is theoretically feasible to design benzoxaborole-based derivatives with larger groups that will target bacteria LRSs but are too big for hcLRS. Indeed, we discovered a compound called ZCL039 that is a potent anti-pneumococcal agent inhibiting *Streptococcus pneumoniae* LRS (*Sp*LRS) activity but with no inhibition of hcLRS activity (35). Compare to tavaborole, an additional benzene ring is added to the meta-C of benzoxaborole (Figure 6C). ZCL039-AMP is well bound in the editing pocket of *Sp*LRS, however, when we superimpose with the structure of hcLRS-CP1, the AMP moiety of ZCL039-AMP could bind well, but the benzene ring of ZCL039 moiety has steric clashes with hcLRS-CP1 (Figure 6G), and the residues that clash with ZCL039 are indeed from the I4ae insertion (Supplementary Figure S8). Taken together, our results showed that it is feasible to design benzoxaborole-based derivatives targeting specifically LRS from bacterial microbes but not hcLRS.

### Assembly of hcLRS into the MSC

**Figure 7.**
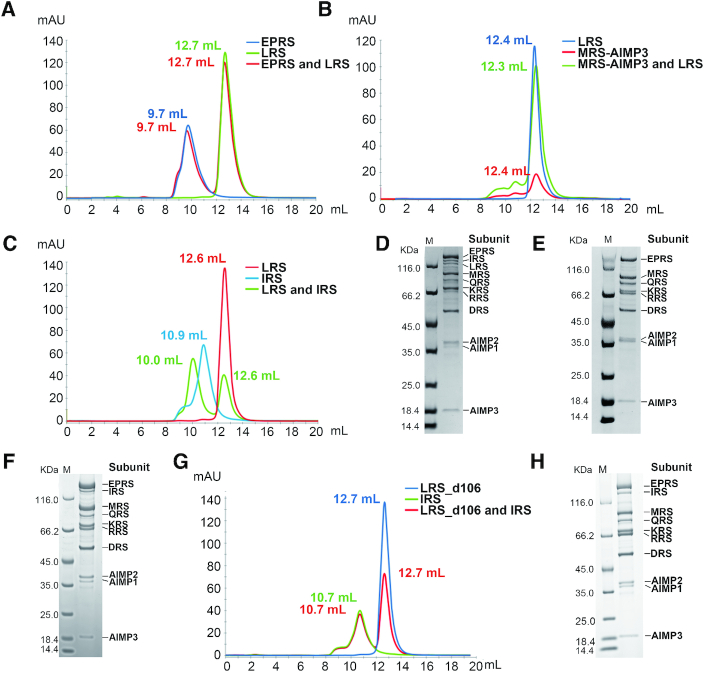
The assembly of hcLRS into the MSC complex. The gel filtration result of hcLRS mixed with EPRS (**A**), or MRS-AIMP3 (**B**), or IRS (**C**), the peak of each component are indicated by the elution volume; The *in vitro* assembly of human MSC complex by all 11 protein components (**D**) or lacking IRS (**E**) or lacking LRS (**F**); Figure G shows the gel filtration result of the hcLRS_d106 and IRS mixture; Figure H shows the *in vitro* assembly of human MSC complex using hcLRS_d106 instead of hcLRS.

To address the question of how hcLRS assembles into the MSC (Supplementary Figure S2), we purified each component of MSC subcomplex I to assay the physical interaction capability of hcLRS between them. Additionally, we assembled the entire MSC complex *in vitro*. We found that hcLRS did not associate with EPRS (Figure 7A), or MRS-AIMP3 (Figure 7B), but could directly interact with IRS (Figure 7C). Interestingly, compared to the *in vitro* assembly of the complete MSC with eleven components (Figure 7D), when devoid of IRS, the *in vitro* assembly of MSC could form but missed both IRS and hcLRS (Figure 7E). When hcLRS was excluded, the MSC could assemble with the remaining 10 components (Figure 7F). These results showed that the assembly of hcLRS into MSC is mediated by direct interaction with IRS. The observation of the direct interaction between hcLRS and IRS is consistent with an earlier study using yeast two-hybrid analysis that suggested that the UNE-L domain of hcLRS interacts with UNE-I domain of IRS (67). To test whether the UNE-L domain of hcLRS is involved in the assembly of MSC, we used hcLRS_d106 with truncation of UNE-L. Intriguingly, we found that hcLRS_d106 completely loses the capacity to bind IRS (Figure 7G) and could not be assembled into the MSC (Figure 7H). Our results clearly show that *in vitro*, hcLRS assembles into the MSC by direct interaction with IRS through the UNE-L domain. *In vivo*, it cannot be ruled out that additional interactions might be involved in MSC assembly and further study is required to fully elucidate the mechanism.

## DISCUSSION

### Functional expansion through recruitments of extra domains/motifs and/or adaptation of already exist domains/motifs

As reported here, hcLRS has a very complex modular architecture. The sequence of LRS become longer and longer during evolution (Supplementary Figure S1), owing to (i) extra sequence inserted in pre-existing domains, such as the long insertion in the LSD1 and the RagD binding peptides; (ii) new domain/motifs appended into LRS, such as UNE-L. Based on current study and previous studies, we proposed that the ancestor of LRS is composed of the Rossmann-fold domain, the ABD domain and the SC-fold. During evolution, extra domains/motifs such as CP1, CP2 and LSD1 domains are recruited to reinforce the efficiency and accuracy of tRNA aminoacylation, the most noticeable one is the recruitment of CP1 which endows LRS to be able to efficiently hydrolyze mischarged-tRNA. Later on, extra domains were inserted or appended to LRSs to further gain additional functions for LRS, such as UNE-L which assembled LRS into the MSC. The most attractive moonlight function of hcLRS is its leucine sensing to modulate the mTORC pathway through direct interaction with RagD. The leucine sensing procedure is performed by the ancient aminoacylation active site, while the binding with RagD is carried out by the later inserted VC-b domain. The sequence of VC (C-ter) domain are less conserved compared to other domains of LRSs (Supplementary Figure S1). Structurally, the C-ter-a (VC-a) domain that is responsible for binding with tRNA is more conserved than the C-ter-b (VC-b) domain. Interestingly, the RagD binding peptide does not share homology with the corresponding region in *Ph*LRS, suggesting that the function for binding with RagD is through adaption of the already exist VC-b domain. The function of other insertions still remains unclear, such as the long low-complexity sequence in LSD1, LSD2 and the four insertions in the CP1 domain.

### Domain orientations and conformational changes

Distinct domain orientations and conformational changes upon substrates binding have been observed in the former structural studies of bacterial and archaeal LRSs (14–17). When we simply superposed the structure of hcLRS onto the *Ph*LRS–tRNA^Leu^ complex, the CP1 domain, the VC-domain and the CP core domain clashed with tRNA^Leu^, suggesting a change in orientation is needed upon tRNA substrate binding. However, the details of how this domain moves require further investigation. In particularly, when sensing leucine, hcLRS binds to RagD. This raises the question as to what kind of conformational change of hcLRS occurs during the switch from tRNA to RagD binding. It is worth noting that structurally disordered regions have been found to mainly play a role in activating and expanding the regulatory functions of aaRSs (55). In hcLRS, the LSD1 domain comprises a long structural disordered peptide and locates close to the KMSKS loop which is important for binding to tRNA. In the LeuAMS bound structure of hcLRS, the nearest distance between the visible part of LSD1 and the KMSKS loop is 6 Å. Moreover, the AMS (AMP) moiety of LeuAMS has wide interactions with the HIGH motif and the KMSKS loop (Figure 2B). Compared with LeuAMS, leucine lacks the AMS part, so it is possible that the KMSKS loop is in another conformation when only leucine is bound. One could imagine that under these conditions, the LSD1 domain could directly interact with the KMSKS loop and that this might inhibit tRNA binding. Hence, it is worth testing in a future study whether the LSD1 plays a role in the switch of hcLRS between tRNA binding and RagD binding.

### Prospects of drugs targeting hcLRS

Tavaborole is used as a topical medication to treat toenail infection caused by a fungus. However, tavaborole is toxic when swallowed (The use and side effects of tavaborole from the U.S. National Library of Medicine: https://medlineplus.gov/druginfo/meds/a614049.html) indicating that it may target either one or both human LRSs. In the current study, we showed that tavaborole inhibits the aminoacylation activity of hcLRS, whereas previously we showed that the human mitochondrial LRS is resistant to tavaborole inhibition due to the degeneration of editing active site in its CP1 domain (68). Taken together, the toxicity of tavaborole likely comes from inhibition of the cytoplasmic hcLRS. Derivatives of tavaborole have great potential in inhibiting pathogenic microbes by targeting their LRS. Our high-resolution structure of hcLRS provides insight into rational optimization of those compounds. The specific I4ae insertions and other divergent residues around the editing active site endow specificity to the editing pocket of hcLRS (Figures 3 and 6D–F). Thanks to these structural differences, it is possible to design compounds that specifically target LRS from pathogens but not hcLRS, ZCL-039 being an example. On the other hand, compounds targeting hcLRS are promising anti-cancer drugs. Whilst our results show the binding details of leucine or LeuAMP analogues in the aminoacylation site, such compounds inhibit the aminoacylation activity of hcLRS at certain concentrations and thus are potentially toxic. In contrast BC-LI-0186 does not affect the binding of tRNA or the aminoacylation activity of hcLRS (31). Although our results show the atomic structure of the potential binding site, complex structure of BC-LI-0186 with hcLRS is still required to further optimize this potential anti-cancer compound.

## DATA AVAILABILITY

Protein Data Bank: atomic coordinates and structure factors for hcLRS_d106 bound with LeuAMS and Nva2AA has been deposited with accession code 6LPF; the accession code for hcLRS_d106 bound with LeuAMS and AN6426-AMP is 6LR6.

## Supplementary Material

gkaa189_Supplemental_File
