# Supplementary material for: Molecular basis of the multifaceted functions of human leucyl-tRNA synthetase in protein synthesis and beyond
Source: Nucleic Acids Res. 2020 Mar 30;48(9):4946–59. doi: 10.1093/nar/gkaa189 (PMC7229842; doi:10.1093/nar/gkaa189)
Supplement: gkaa189_Supplemental_File [file gkaa189_supplemental_file.pdf]

Supplementary tables and figures:

Table S1. Data collection and refinement statistics

**Measurement**	**HcLRS_d106 + LeuAMS + Nva2AA**	**HcLRS_d106 + LeuAMS + AN6426-AMP**
**Data collection**		
Space group	$C222_1$	$C222_1$
Cell dimensions		
a, *b*, *c* (Å)	88.21 94.68 680.05	88.85 95.79 682.38
α, β, γ (°)	90 90 90	90 90 90
Resolution (Å) [a]	50-3.00 (2.54-2.50)	50-3.00 (3.11-3.00)
R_{linear}	0.088 (0.704)	0.116 (0.539)
$I / \sigma I$	26.4 (2.4)	9.4 (2.3)
Completeness (%)	99.9 (98.6)	77.7 (74.2)
Redundancy	9.5 (6.2)	4.1 (3.9)
**Refinement**		
Resolution (Å)	50-2.50	50-3.0
No. reflections (work/free)	97695/4888	43749/2242
R_{work} / R_{free}	0.198/0.242	0.211/0.261
No. atoms		
Protein	16212 (2 chains)	16210 (2 chains)
Water	682	10
Ligand		
LeuAMS	62 (2 molecules)	62 (2 molecules)
Nva2AA	26 (1molecule)	
AN6426-AMP		76 (2 molecules)
B-factors		
Protein	65.5 (Chain A); 34.5(Chain B)	79.3(Chain A); 39.1(Chain B)
water	36.3	20.9
Ligand		
LeuAMS	46.1	36.1
Nva2AA	40.2 (Chain B)	
AN6426-AMP		59.4 (Chain B)
Bond lengths (Å)	0.004	0.003
Bond angles (°)	0.632	0.725

[a] Values in parentheses are for the highest-resolution shell.

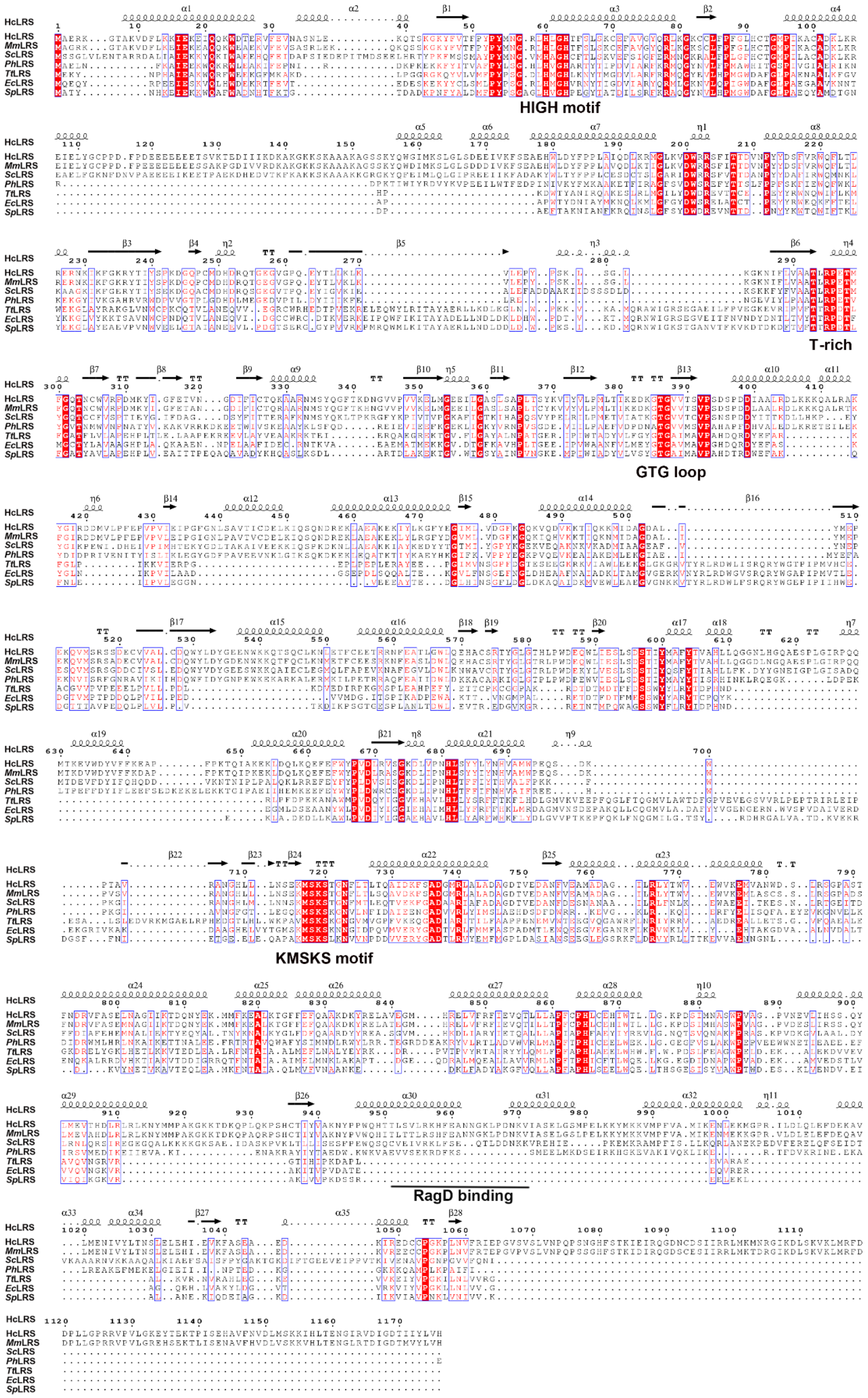
HcLRS
MmLRS
ScLRS
PhLRS
TtLRS
EcLRS
SpLRS
HIGH motif
T-rich
GTG loop
KMSKS motif
RagD binding

Figure S1. Structure based sequence alignment of hcLRS with LRSs from representative species. The secondary structure elements of hcLRS are labeled in the top, and the conserved motifs of LRSs are marked in the bottom.

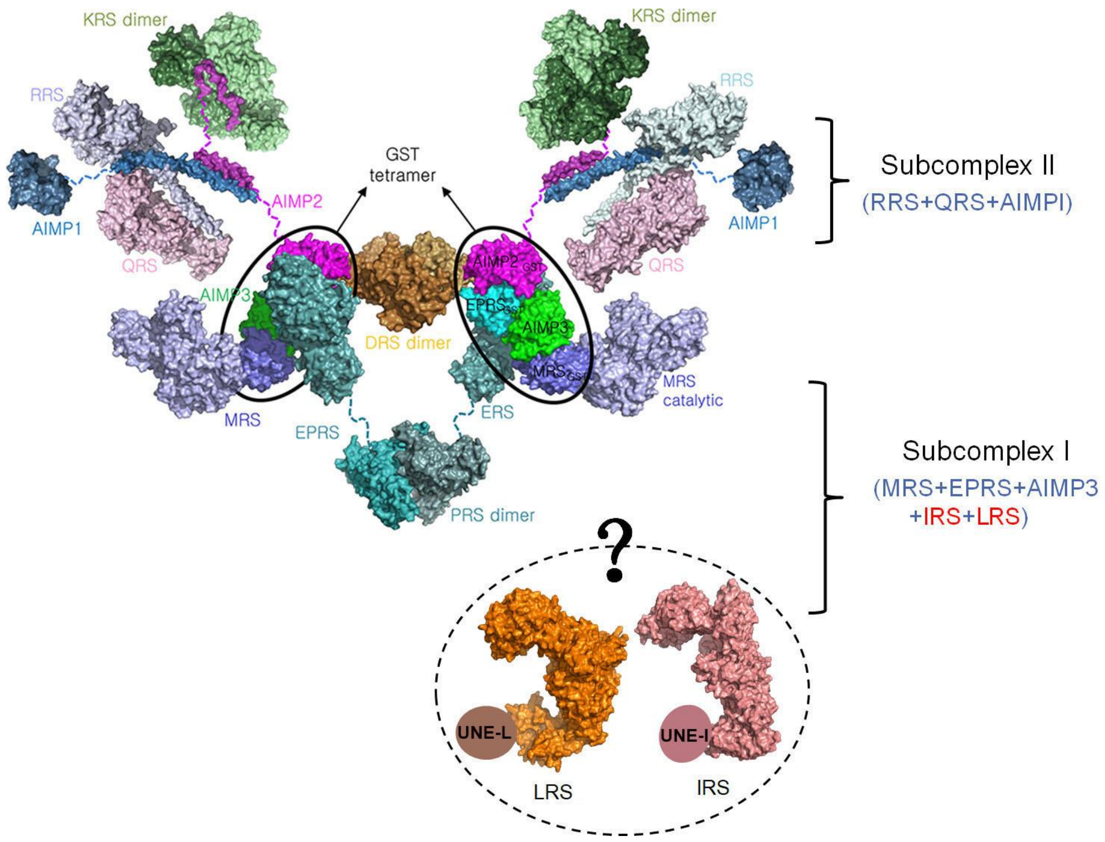

Figure S2. The model of MSC adapted from Cho et al, 2015. The surface model of LRS is from this study, and the surface model of IRS is generated based on the structure of bacterial IRS (PDB: 1FFY). The structures of UNE-L or UNE-I are unknown.

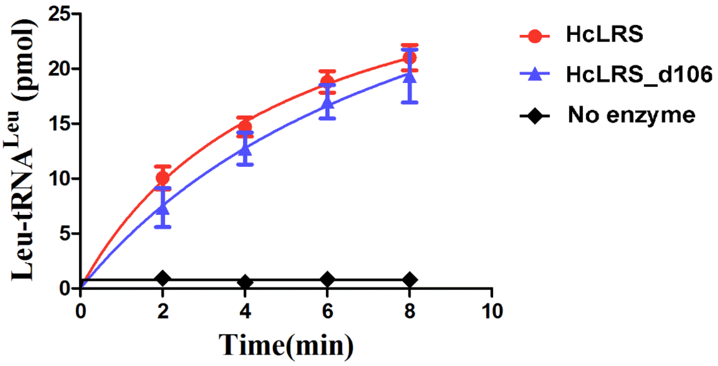

Figure S3. Diagram of the aminoacylation activity of full length hcLRS and hcLRS_d106.

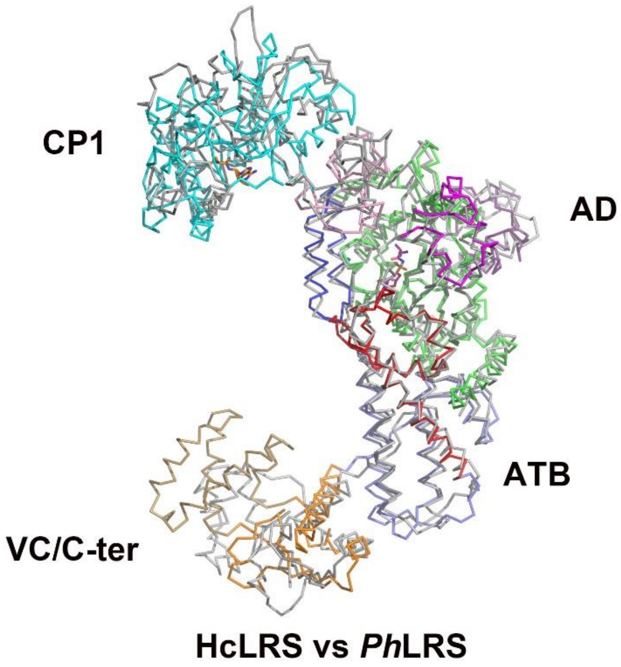

Figure S4. Superposition of hcLRS (colors are identical with Figure 1A) to *Ph*LRS (light grey, only the protein part from PDB: 1WZ2) shown in ribbon.

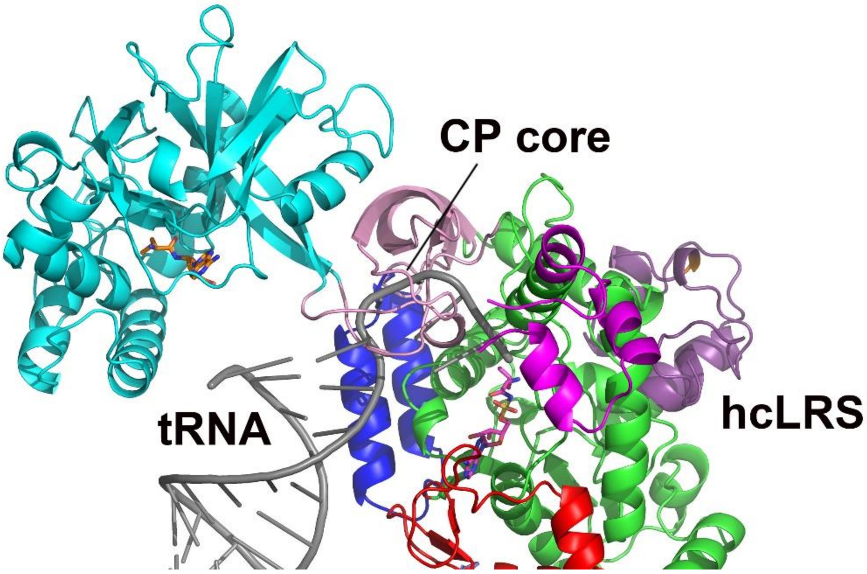

Figure S5. Superposition of hcLRS (colors are identical with Figure 1A) to the *Ph*LRS-tRNA complex (PDB: 1WZ2, only show the tRNA part). The CP core clashes with 3'-end of tRNA.

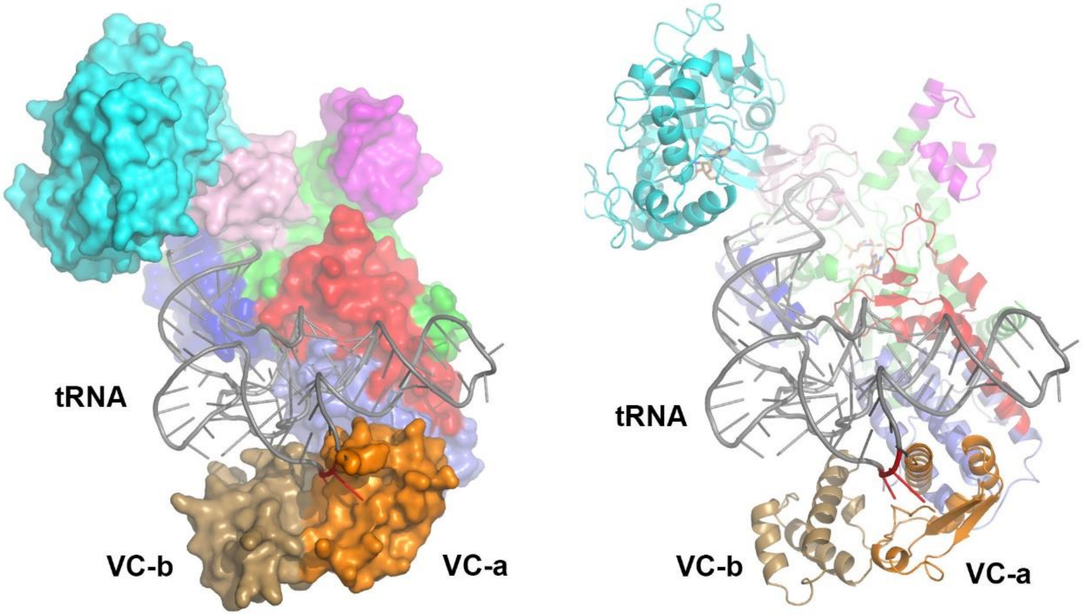

Figure S6. The proposed docking model of hcLRS-tRNA^{Leu} complex. HcLRS is shown in surface (left) or in cartoon (right), the colors of each domain and motif are the same as those in Figure 1A, tRNA^{Leu} is shown in grey. Two residues from the long variable loop of tRNA^{Leu} that contact with VC domain are marked in red color.

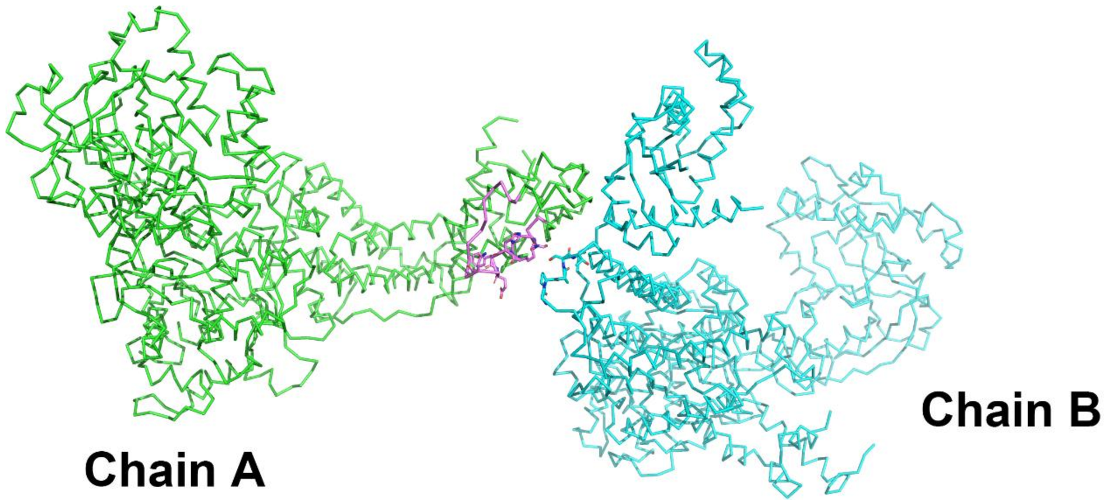

Figure S7. Ribbon diagram shows the crystal packing interface of hcLRS. The residues from the RagD binding peptide (magenta) of Chain A, and those that are interact with residues in the interface are shown in sticks.

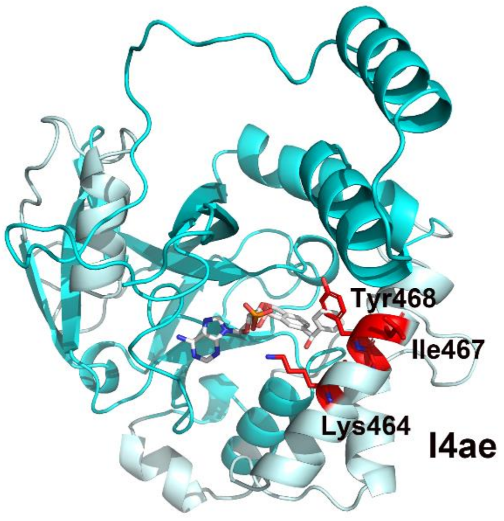

Figure S8. Docking model of ZCL039-AMP (Shown in sticks with backbone in white color) into the editing site of hcLRS by superimposing the structure of hcLRS-CP1 to *Sp*LeuRS-CP1-ZCL039-AMP complex (PDB: 4K47). The ZCL039 moiety has steric clashes with residues (red color) from the I4ae insertion of hcLRS.
